# Quackery and the Newspaper Press

**Published:** 1847-08

**Authors:** 


9. *Quackery and the Newspaper Press.*—In Scotland the presses are beginning, in real earnest, to oppose the vile system of quack medicines and quack advertisements. Articles have been forwarded to us from Chambers' Edinburgh Journal, the Scottish Reformers Gazette, the Glasgow Constitutional, the Fifeshire Journal, and other respectable newspapers, expressing their determined opposition to medical quackery in every shape. The Glasgow Constitutional tersely observes:—

"We have often been astonished that some journals, otherwise as respectable as their neighbors, should, for any trifling pecuniary advantage, place themselves in the position of *socii criminis* to a parcel of vagabond quacks.

"We consider the insertion of quack advertisements a most dangerous imposition, and the persons who give it as little better than the more daring criminal. The quack and his newspaper agent are as necessary to each other, in order to dupe the unwary with complete success, as is the receiver to the thief, being all in concert; and they divide the spoil wrung from the pallid hand of poverty, disease, and death."

Chambers' Edinburgh Journal, entering, as it does, the homes of tens of thousands in every part of the empire, can do immense service to the anti-quackery cause. In Ireland, also, the anti-quackery feeling is alive. As we stated some weeks ago, the Nation is most vigorous and determined in its reprobation of quack medicines and advertisements. Side by side with this paper we must place the Dublin General Advertiser. We are sanguine in the hope, that the press which has so long been the strong hold of quackery, will now become an agent in its destruction. We contend that every qualified medical man should become an active propagandist of anti-quackery opinion, above all, should endeavor to influence the public press for the suppressing of the whole system.—*Lancet.*

The profession could do much to abate this monstrous evil, if they would exert the power they possess. Let them exert their influence in favor of those papers which refuse insertion to quack advertisements, and discountenance those which publish such advertisements, and the unholy alliance which now exists between quackery and the newspaper press would be broken up.—*Med. News.*

